# External validation of the modified Marsh and Schnider models for medium-chain triglyceride propofol in target-controlled infusion anesthesia

**DOI:** 10.1186/s12871-024-02461-5

**Published:** 2024-02-23

**Authors:** Seongheon Lee, Dongho Kang, Eunjin Song, Sungah Yoo, Seongwook Jeong

**Affiliations:** 1 Department of Anesthesiology and Pain Medicine, Chonnam University Hospital, Gwangju, South Korea; 2 Department of Anesthesiology and Pain Medicine, Chonnam National University Medical School, 160, Baekseo-ro, Dong-gu, Gwangju, South Korea

**Keywords:** Propofol formulation, Schnider model, Marsh model, Target controlled infusion, External validation

## Abstract

**Background:**

Propofol formulated with medium- and long-chain triglycerides (MCT/LCT propofol) has rapidly replaced propofol formulated with long-chain triglycerides (LCT propofol). Despite this shift, the modified Marsh and Schnider pharmacokinetic models developed using LCT propofol are still widely used for target-controlled infusion (TCI) of propofol. This study aimed to validate the external applicability of these models by evaluating their predictive performance during TCI of MCT/LCT propofol in general anesthesia.

**Methods:**

Adult patients (*n* = 48) undergoing elective surgery received MCT/LCT propofol via a TCI system using either the modified Marsh or Schnider models. Blood samples were collected at various target propofol concentrations and at specific time points, including the loss of consciousness and the recovery of consciousness (13 samples per patient). The actual plasma concentration of propofol was determined using high-performance liquid chromatography. The predictive performance of each pharmacokinetic model was assessed by calculating four parameters: inaccuracy, bias, divergence, and wobble.

**Results:**

Both the modified Marsh and Schnider models demonstrated predictive performances within clinically acceptable ranges for MCT/LCT propofol. The inaccuracy values were 24.4% for the modified Marsh model and 26.9% for the Schnider model. Both models showed an overall positive bias, 16.4% for the modified Marsh model and 16.6% for the Schnider model. The predictive performance of MCT/LCT propofol was comparable to that of LCT propofol, suggesting formulation changes might exert only a minor impact on the reliability of the TCI system during general anesthesia. Additionally, both models exhibited higher bias and inaccuracy at target concentrations ranging from 3.5 ~ 5 ug/ml than at concentrations between 2 ~ 3 ug/ml.

**Conclusions:**

The modified Marsh and Schnider models, initially developed for LCT propofol, remain clinically acceptable for TCI with MCT/LCT propofol.

**Trial registration:**

This study was registered at the Clinical Research Information Service of the Korean National Institute of Health (https://cris.nih.go.kr; registration number: KCT0002191; 06/01/2017).

## Background

Propofol is the most widely-used intravenous anesthetic agent in contemporary medical practice. Traditionally, propofol has been formulated in a fat emulsion consisting of long-chain triglyceride (LCT) [1]. However, this formulation often leads to a high incidence of moderate to severe pain on injection. To solve this problem, a new formulation of propofol containing a combination of 50% medium-chain triglyceride (MCT) and 50% LCT was introduced. This MCT/LCT emulsion reduces the concentration of free propofol in the aqueous phase of the emulsion, which is associated with injection pain. Due to its advantage in causing less injection pain, MCT/LCT propofol has rapidly replaced LCT propofol.

Modifying the composition of the carrier emulsion for propofol might influence the pharmacokinetics or pharmacodynamics of the drug [2–4]. Some earlier studies indicated that the pharmacokinetic parameters calculated were not markedly different between LCT and MCT/LCT emulsion following a single intravenous injection or a short-term infusion in volunteers [5, 6]. Conversely, Le Guen et al. found that considerable differences in potency between propofol formulations could be observed during general anesthesia when using a target-controlled infusion (TCI) system for surgical patients [7]. Different models might deliver different amounts of propofol [8]. Most popular propofol models in the commercialized TCI pump system, such as the modified Marsh and Schnider models, were developed for LCT propofol, not MCT/LCT propofol. If there is a meaningful difference in TCI performance between LCT and MCT/LCT propofol, it may necessitate the development of a new model specifically for MCT/LCT propofol.

The predictive performance of a pharmacokinetic model refers to how accurately it can predict the plasma concentrations of a drug based on factors such as dosage, time since administration, and individual characteristics. Although the time course of propofol concentrations with limited aqueous solubility can be altered by changing the lipid emulsion formulation, there has been no investigation of the predictive performance of popular propofol models by directly measuring plasma concentrations in situations when MCT/LCT propofol is infused for a long-time anesthesia using TCI. Hence, it remains unclear whether the modified Marsh and Schnider models are suitable for TCI with MCT/LCT propofol. This research aimed to determine the predictive performances of the modified Marsh and Schnider models during general anesthesia with TCI using MCT/LCT propofol, without making a direct comparison between LCT and MCT/LCT propofol.

## Methods

This study was approved by the Institutional Review Board of Chonnam National University Hwasun Hospital (IRB no. CNUHH-2014–101) and was registered at the Clinical Research Information Service of the Korean National Institute of Health (https://cris.nih.go.kr; registration number: KCT0002191; 06/01/2017). Written informed consent was obtained from all participants before enrollment.

### Patient population

This study enrolled adult patients with BMI < 30 kg/m^2^ and an American Society of Anesthesiologists physical status classification of I-II who were scheduled to receive general anesthesia for elective abdominal surgery. Exclusion criteria were hemoglobin < 10 g/dl, anticipated blood loss exceeding 500 mL during surgery, or a history of hematologic, renal, neurologic, or psychiatric diseases.

Patients (*n* = 48) were randomized into two groups: the modified Marsh model group and the Schnider model group. Randomization was stratified by gender and age (20–40, 41–64, ≥ 65 years). Group allocation within each stratum was based on a computer-generated random number list with permuted blocks. Each patient received MCT/LCT propofol (Freefol‐MCT®; Daewon Pharmaceutical Co Ltd, Seoul, South Korea) via the Orchestra Base Primea® (Fresenius Kabi, France) TCI system using the modified Marsh or Schnider model according to the allocated group.

### Anesthesia and blood sampling

Upon arrival in the operating room without premedication, patients were monitored using electrocardiography, non-invasive blood pressure, pulse oximetry, capnography, and bispectral index (BIS Vista, Medtronic). An angiocatheter was placed in the radial artery for periodic blood sampling and continuous blood pressure monitoring. Anesthesia induction began with an initial propofol target effect site concentration (Ce) of either 3.0 or 3.5 ug/ml. Loss of consciousness (LOC) was determined by patient responsiveness to simple verbal commands. After LOC, remifentanil was administered with an initial target Ce of 3.0 ng/ml via a TCI system using the Minto model. Target Ce of remifentanil was adjusted during anesthesia to maintain blood pressure and heart rate between 80 and 120% of baseline values. Tracheal intubation was facilitated by rocuronium.

The initial target Ce for anesthesia induction was maintained for 10 min. According to the study protocol, the target Ce of propofol was subsequently changed to 4, 5, 4, 3, and 2.5 ug/ml during surgery, maintaining each target for at least a 10-min interval. When peritoneal closure was begun, the target Ce of propofol was changed to 2.0 or 2.2 ug/ml. At the end of surgery, propofol and remifentanil were stopped, and pyridostigmine and glycopyrrolate were used for reversal of the neuromuscular blockade. Recovery of consciousness (ROC) was determined by the recovery of response to verbal commands.

Arterial blood samples (3 ml) were collected after waiting for 10 min following the start or any change of the target Ce. The last blood sample was obtained an hour after propofol discontinuation in the post-anesthesia care unit. These samples were used to evaluate the predictive performance of each model. Additionally, extra blood samples were collected at the moment of LOC and ROC to determine the concentrations at these specific points. These extra samples were not included in the predictive performance calculations.

### Plasma concentration assay

Plasma propofol concentrations were quantified using a high-performance liquid chromatography-tandem mass spectrometry (LC–MS/MS). Briefly, each blood sample was collected in an ethylenediamine tetraacetic acid (EDTA) tube and centrifuged for 10 min at 1500 g; the resultant plasma was stored at -70 ℃ until required for the assay. Plasma concentrations of propofol were analyzed using ultrafast lipid chromatography (Shimadzu, Kyoto, Japan) coupled with tandem mass spectrometry (API5500, SCIEX, Framingham, MA, USA). An ACE 5 C18 column was used for chromatographic separation. The mobile phase consisted of a mixture of water and methanol (30:70, v/v), and a flow rate of 0.35 ml/min was used. The column oven temperature was maintained at 40 ℃, and the injection volume was 4 ul. The validated quantification range was 50 ~ 10,000 ng/ml. The precision (coefficients of variation) and accuracy (relative errors of the mean) of intra- and inter-day analyses were verified to be within 15% and 85–115%, respectively.

### Performance analysis

The predictive performance of each pharmacokinetic model was assessed via the calculation of four universal parameters: inaccuracy, bias, divergence, and wobble [9]. Individual parameters for predictive performance were calculated using specific equations as follows. First, performance error (PE) was calculated as how close the measured concentration (Cm) is to the predicted concentration (Cp) at the j_th_ sampling point from the i_th_ patient:

$${{\text{PE}}}_{{\text{ij}}}(\mathrm{\%})=\frac{{{\text{Cm}}}_{{\text{ij}}}-{{\text{Cp}}}_{{\text{ij}}}}{{{\text{Cp}}}_{{\text{ij}}}}\times 100$$

The ‘inaccuracy’ of the model was calculated as the median absolute PE (MDAPE_i_), presenting the size of the performance errors:

$${{\text{MDAPE}}}_{{\text{i}}} \left(\mathrm{\%}\right)=\mathrm{median }\left\{\left|{{\text{PE}}}_{{\text{ij}}}\right|,\mathrm{ j}=1, \dots , {{\text{N}}}_{{\text{i}}}\right\}$$

where N_i_ is the number of blood sampling points for the i_th_ patient.

The ‘bias’ of the model was calculated as the median PE (MDPE_i_), presenting the direction of the performance errors:

$${{\text{MDPE}}}_{{\text{i}}} \left(\mathrm{\%}\right)=\mathrm{median }\left\{{{\text{PE}}}_{{\text{ij}}},\mathrm{ j}=1, \dots , {{\text{N}}}_{{\text{i}}}\right\}$$

The ‘divergence’ of the model was defined as the slope of linear regression of absolute PE values against time, presenting time-related trend of performance errors:

$${{\text{Divergence}}}_{{\text{i}}} (\mathrm{\%}/{\text{h}})=60\times \frac{{\sum }_{{\text{j}}=1}^{{{\text{N}}}_{{\text{i}}}}\left|{{\text{PE}}}_{{\text{ij}}}\right|\times {{\text{t}}}_{{\text{ij}}}-\left({\sum }_{{\text{j}}=1}^{{{\text{N}}}_{{\text{i}}}}\left|{{\text{PE}}}_{{\text{ij}}}\right|\right)\times \left({\sum }_{{\text{j}}=1}^{{{\text{N}}}_{{\text{i}}}}{{\text{t}}}_{{\text{ij}}}\right)/{{\text{N}}}_{{\text{i}}}}{{\sum }_{{\text{j}}=1}^{{{\text{N}}}_{{\text{i}}}}({{\text{t}}}_{{\text{ij}}}{)}^{2}-{\left({\sum }_{{\text{j}}=1}^{{{\text{N}}}_{{\text{i}}}}{{\text{t}}}_{{\text{ij}}}\right)}^{2}/{{\text{N}}}_{{\text{i}}}}$$

where t_ij_ is the time (min) when the corresponding PE_ij_ was determined. High divergence value is related to the widening tendency of the gap between measured and predicted concentrations over time.

The ‘wobble’ is calculated as the median absolute deviation of PE from MDPE, presenting the intra-individual variability of performance errors:

$${{\text{Wobble}}}_{{\text{i}}} \left(\mathrm{\%}\right)=\mathrm{median }\left\{{\left|{{\text{PE}}}_{{\text{ij}}}-{{\text{MDPE}}}_{{\text{i}}}\right|},\mathrm{ j}=1, \dots , {{\text{N}}}_{{\text{i}}}\right\}$$

Low wobble value is related to the ability to maintain stable drug concentrations.

After calculating all parameters including inaccuracy, bias, divergence, and wobble for each patient, population estimates of the parameters were calculated by a pooled data approach using fit4NM program (http://www.fit4nm.org).

### Statistical analysis

Statistical analyses were conducted using R (version 4.2.3, R Foundation for Statistical Computing, Vienna, Austria). The Kolmogorov–Smirnov test was performed to examine the assumption of normality. Normally distributed continuous variables were compared using the student’s t-test; non-normally distributed continuous variables and ordinal variables were compared using the Mann–Whitney U test. Categorical variables were compared using the chi-square test or Fisher’s exact test. Data are expressed as the number of patients, mean ± standard deviation, or median (95% confidence interval). A *P*-value below 0.05 was considered statistically significant.

## Results

This study initially aimed to collect 624 arterial blood samples from 48 patients (13 samples per patient). However, 11 samples were not collected due to unexpected short surgery durations (*n* = 3), arterial catheter malfunctions (*n* = 4), and technical errors (*n* = 4). As a result, 613 plasma samples from 48 patients were analyzed for propofol concentrations: 309 from the Marsh group, 304 from the Schnider group). The demographic data showed no significant differences between the groups (Table 1). The mean infusion durations were 236.3 min for the Marsh group and 220.6 min for the Schnider group.

**Table 1:** Demographic data

Characteristics	Modified Marsh model (*n* = 24)	Schnider model (*n* = 24)
**Male / Female**	12 / 12	12 / 12
**Age (yr)**	54.0 ± 12.9	54.0 ± 13.4
**Weight (kg)**	62.3 ± 9.7	62.6 ± 9.0
**Height (cm)**	161.8 ± 8.1	161.9 ± 8.6
**BMI (kg/m**^**2**^**)**	23.7 ± 3.1	23.8 ± 2.2
**Propofol TCI time (min)**	236.3 ± 76.1	220.5 ± 67.5

Data are presented as number of patients or mean ± SD

When MCT/LCT propofol was administered using either the modified Marsh or Schnider models, the predictive performances of the two models were comparable in terms of pooled bias, inaccuracy, divergence, and wobble (Table 2). The overall bias (MDPE) was 16.4% for the modified Marsh model and 16.6% for the Schnider model. These positive biases indicate that both models could underpredict plasma concentrations during TCI situations. While the 95% confidence interval for MDPE did not include zero in both models, suggesting significant biases, the overall bias (MDPE) was < 20% and inaccuracy (MDAPE) was < 30%. These results are within clinically acceptable range, as described in previous studies [10–12].

**Table 2:** Pooled biases (MDPE), inaccuracies (MDAPE), divergences, and wobbles (with 95% confidence intervals) during TCI of propofol formulated with medium- and long-chain triglycerides using the modified Marsh and Schnider models

	**Target concentrations**	**Modified Marsh Model**	**Schnider Model**
**Bias (%), MDPE**	Overall	16.4 (12.7 to 20.1)^a^	16.6 (13.2 to 20.2)^a^
	2 ~ 3 ug/ml	4.4 (0.02 to 8.8)	6.8 (1.8 to 11.9)
	3.5 ~ 5 ug/ml	27.7 (24.8 to 30.7)	24.5 (21.8 to 27.2)
**Inaccuracy (%), MDAPE**	Overall	24.4 (21.8 to 26.9)	26.9 (23.9 to 29.6)
	2 ~ 3 ug/ml	19.5 (16.9 to 22.1)	24.0 (19.9 to 28.0)
	3.5 ~ 5 ug/ml	31.6 (28.8 to 34.4)	29.5 (27.1 to 32.0)
**Divergence (%/h)**	Overall	-3.3 (-4.7 to -1.8)	-7.2 (-11.8 to -6.0)
	2 ~ 3 ug/ml	0.0 (-1.5 to 1.5)	-5.6 (-7.6 to -3.7)
	3.5 ~ 5 ug/ml	-9.0 (-18.4 to 0.4)	11.6 (2.4 to 20.8)
**Wobble (%)**	Overall	11.7 (9.1 to 14.3)	8.8 (5.9 to 11.4)
	2 ~ 3 ug/ml	12.2 (9.3 to 15.2)	12.8 (10.2 to 15.4)
	3.5 ~ 5 ug/ml	6.3 (4.3 to 8.2)	4.9 (3.0 to 6.7)

*MDPE* Median performance error, *MDAPE* Median absolute performance error, *TCI* Target controlled infusion

^a^Significant bias: 95% confidence interval of MDPE did not include zero

**Fig. 1:**
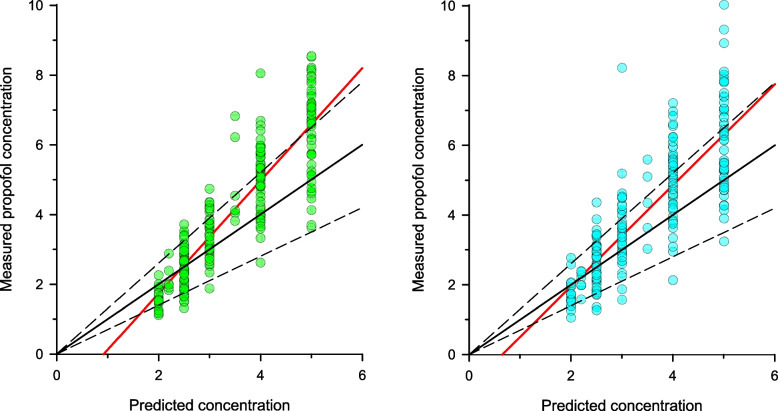
Measured vs. predicted plasma concentration of propofol for the modified Marsh model (left) and Schnider model (right). The black solid and dashed lines represent the line of identity and biases of ± 30%, respectively. The red solid lines indicate the linear regression between measured and predicted values

Both models exhibited higher bias and inaccuracy at target concentrations ranging from 3.5 ~ 5 ug/ml than at concentrations between 2 ~ 3 ug/ml (Table 2). The measured vs. predicted plasma concentration plots also shows the tendency that the measured concentrations are generally higher than the predicted concentrations (underprediction) when the predicted concentration is above 3.0 ug/ml (Fig. 1). The overall divergence and wobble associated with the predictions of both models were comparable with those found in previous studies [10, 13].

Table 3 presents the measured plasma concentrations of propofol during induction and recovery phase. During the induction phase, an initial target Ce was 3.0 or 3.5 ug/ml in both models. Notably, the concentration at LOC for the modified Marsh model was much higher than the target Ce. This temporary overshoot of the plasma concentration in the modified Marsh model resulted in a more rapid LOC compared to the Schnider model (0.97 vs. 5.03 min). In contrast, the concentration at LOC for the Schnider model was slightly below the target Ce. Nevertheless, 10 min after starting TCI, concentrations in both models reached similar levels, marginally above the target Ce. During the recovery phase, the concentrations were comparable between the two models.

**Table 3:** Measured propofol concentrations during induction and recovery phases including at LOC and ROC times

Period	Measured plasma concentration (Cp, ug/ml) and time (min)	Modified Marsh model (*n* = 24)	Schnider model (*n* = 24)	*P* value
**Induction phase**	Cp at LOC	9.88 ± 7.00	2.65 ± 0.83	< 0.001
	LOC time from TCI start	0.97 ± 0.22	5.03 ± 3.77	< 0.001
	Cp at 10 min after TCI start	3.83 ± 1.00	3.75 ± 1.27	0.808
**Recovery phase**	Cp at TCI stop	1.73 ± 0.41	1.85 ± 0.44	0.342
	Cp at ROC	0.96 ± 0.23	0.97 ± 0.24	0.861
	ROC time from TCI stop	7.03 ± 5.83	7.88 ± 4.62	0.578
	Cp at 60 min after TCI stop	0.59 ± 0.15	0.58 ± 0.13	0.879

Data are presented as mean ± SD. During induction phase, an initial target Ce was 3.0 or 3.5 ug/ml and was not changed until 10 min after TCI start (Ce 3.5 for 6 patients for each model). At the end of surgery, TCI was stopped at Ce 2.0 or 2.2 ug/ml (Ce 2.2 for 6 patients for each model)

*LOC* Loss of consciousness, *ROC* Recovery of consciousness, *TCI* Target controlled infusion, *Ce* Effect-site concentration

## Discussion

Accessing the broad applicability of a predictive model is crucial. A significant part of this assessment involves examining the model using data distinct from the data used in its development. In our study, we externally validated the modified Marsh and Schnider models using real concentration data obtained from patients undergoing major surgery with TCI of MCT/LCT propofol. It has been suggested that for a TCI system to be clinically acceptable, the MDPE should not exceed 10–20% and the MDAPE should be between 20–30% [12]. Such error ranges haven’t shown an increased rate of adverse events during TCI [14]. Even though both popular propofol models were developed for LCT propofol, our findings show that their predictive performances remain within clinically acceptable ranges for MCT/LCT propofol.

For effective and safe anesthesia using TCI with pharmacokinetic models for propofol, the actual plasma concentration of propofol should be stably maintained closed to the target plasma concentration. This capability of a pharmacokinetic model has been evaluated by predictive performance, including four parameters of bias, inaccuracy, divergence, and wobble. Glen et al. assessed the predictive performance of various propofol models for LCT propofol using simulated plasma concentration values during TCI [15]. Their results revealed MDAPEs of 26% for the modified Marsh model and 23% for the Schnider model. Those values were within the clinically acceptable range of 20–30%. The overall MDAPEs for MCT/LCT propofol in the present study (24.4% for the modified Marsh model and 26.9% for the Schnider model) are comparable to the results for LCT propofol and are clinically acceptable for both models.

Concerning MDPE, both models showed overall positive biases (where the measured concentration > predicted), which are also within clinically acceptable ranges (16.4% for modified Marsh model and 16.6% for Schnider model). The difference in lipid formulation does not seem to be responsible for this significant bias, because previous studies for LCT propofol also reported similar MDPE values for both models (16% and 15%, respectively).

MCT/LCT emulsion has some different characteristics compared to LCT emulsion. For instance, propofol in MCT/LCT emulsion can be attributed to the reduced amount of propofol in the aqueous phase [16], and MCT undergo lipolysis about twice as fast as LCT [17]. However, the predictive performance from long-duration TCI of MCT/LCT propofol in the present study was not different from previous results for LCT propofol [15]. Despite the differences in propofol formulations, the active component remains the same. Thus, the formulation change might have minimal impact on propofol clearance, which determines the sustained infusion rate in TCI. In a previous study comparing the pharmacokinetic properties of LCT propofol and microemulsion propofol, clearances of the two formulations were similar, at 1.55 L/min and 1.53 L/min, respectively [4].

For the same active component, pharmacokinetics of different formulations might be mainly affected by the distribution volume. Dutta et al. demonstrated that administration of propofol in a lipid-free vehicle led to a 10-fold increase in the central distribution volume compared to lipid emulsion propofol [2]. However, our study suggests that not only clearance but also the volume of distribution might not differ markedly between LCT/MCT propofol and LCT propofol, as shown in previous pharmacokinetic studies [5, 6].

Interestingly, we observed a remarkable difference in bias based on the level of target concentration in our study. At lower target concentrations of 2 ~ 3 ug/ml, both the modified Marsh and Schnider models exhibited acceptable biases. However, at concentrations exceeding 3 ug/ml, a more pronounced positive bias was observed in both models. This positive bias occurs when the measured concentration exceeds the predicted concentration, suggesting an underprediction by the model. The greater bias at higher target concentrations could be attributed to the fundamental characteristics of pharmacokinetic models, where prediction errors typically increase in proportion to the concentration. Additionally, high concentrations may exacerbate individual pharmacokinetic differences, which are influenced by physiological factors such as liver function or cardiac output, resulting in variability in metabolic rates.

When the concentration of a drug rapidly changes, as during induction or recovery phase of anesthesia, the pattern of bias can vary among pharmacokinetic models [15]. In the present study, the LOC time in the Schnider model was more prolonged than in the modified Marsh model (5.03 min vs. 0.97 min) (Table 3). The faster LOC time in the modified Marsh model can be attributed to overshooting of the plasma concentration, which was evident throughout a much higher concentration than the target concentration at LOC. In contrast, the delayed LOC time in the Schnider model was due to negative bias, as observed from the lower concentration than the target concentration at LOC. Low fixed volume of distribution in the central compartment, as a characteristic of the Schnider model, seems to contribute the negative bias (overprediction) during the induction phase [15]. Administrating an induction dose into this limited volume of distribution leads to a high concentration prediction.

This study has several limitations. Firstly, the absence of a control group using LCT propofol limited our ability to compare the predictive performance between LCT and MCT/LCT formulations. However, the predictive performance of both the modified Marsh and Schnider models was found to be within clinically acceptable ranges when using MCT/LCT propofol. Thus, our findings still provide meaningful insights into the application of these models with MCT/LCT propofol. Secondly, our evaluation focused on older pharmacokinetic models, particularly the modified Marsh and Schnider models, rather than newer second-generation models like the Eleveld model. However, the results of this study lead us to believe that differences in formulation between LCT an MCT/LCT propofol may not significantly impact the predictive performance of other propofol models.

## Conclusions

In conclusion, the predictive performances of both the modified Marsh and Schnider model are clinically acceptable. It can be judged that TCI of MCT/LCT propofol using these two popular models on a commercialized TCI system is scientifically valid.

## Data Availability

The datasets used and/or analysed during the current study are available from the corresponding author on reasonable request.

## Abbreviations

LCT: Long-chain triglyceride
MCT: Medium-chain triglyceride
TCI: Target-controlled infusion
Ce: Effect site concentration
LOC: Loss of consciousness
ROC: Recovery of consciousness
LC–MS/MS: Liquid chromatography-tandem mass spectrometry
EDTA: Ethylenediamine tetraacetic acid
PE: Performance error
Cm: Measured concentration
Cp: Predicted concentration
MDAPE: Median absolute performance error
MDPE: Median performance error

## References

[1] Baker MT, Naguib M (2005). Propofol: the challenges of formulation. Anesthesiology.

[2] Dutta S, Ebling WF (1998). Formulation-dependent pharmacokinetics and pharmacodynamics of propofol in rats. J Pharm Pharmacol.

[3] Cox EH, Knibbe CA, Koster VS, Langemeijer MW, Tukker EE, Lange R (1998). Influence of different fat emulsion-based intravenous formulations on the pharmacokinetics and pharmacodynamics of propofol. Pharm Res.

[4] Kim KM, Choi BM, Park SW, Lee SH, Christensen LV, Zhou J (2007). Pharmacokinetics and pharmacodynamics of propofol microemulsion and lipid emulsion after an intravenous bolus and variable rate infusion. Anesthesiology.

[5] Ward DS, Norton JR, Guivarc’h PH, Litman RS, Bailey PL (2002). Pharmacodynamics and pharmacokinetics of propofol in a medium-chain triglyceride emulsion. Anesthesiology.

[6] Doenicke AW, Roizen MF, Rau J, O’Connor M, Kugler J, Klotz U (1997). Pharmacokinetics and pharmacodynamics of propofol in a new solvent. Anesth Analg.

[7] Le Guen M, Grassin-Delyle S, Cornet C, Genty A, Chazot T, Dardelle D (2014). Comparison of the potency of different propofol formulations: a randomized, double-blind trial using closed-loop administration. Anesthesiology.

[8] Absalom AR, Mani V, De Smet T, Struys MM (2009). Pharmacokinetic models for propofol–defining and illuminating the devil in the detail. Br J Anaesth.

[9] Varvel JR, Donoho DL, Shafer SL (1992). Measuring the predictive performance of computer-controlled infusion pumps. J Pharmacokinet Biopharm.

[10] Glen JB, Servin F (2009). Evaluation of the predictive performance of four pharmacokinetic models for propofol. Br J Anaesth.

[11] Vuyk J, Engbers FH, Burm AG, Vletter AA, Bovill JG (1995). Performance of computer-controlled infusion of propofol: an evaluation of five pharmacokinetic parameter sets. Anesth Analg.

[12] Schuttler J, Kloos S, Schwilden H, Stoeckel H (1988). Total intravenous anaesthesia with propofol and alfentanil by computer-assisted infusion. Anaesthesia.

[13] Mertens MJ, Engbers FH, Burm AG, Vuyk J (2003). Predictive performance of computer-controlled infusion of remifentanil during propofol/remifentanil anaesthesia. Br J Anaesth.

[14] Schnider TW, Minto CF, Struys MM, Absalom AR (2016). The safety of target-controlled infusions. Anesth Analg.

[15] Glen JB, White M (2014). A comparison of the predictive performance of three pharmacokinetic models for propofol using measured values obtained during target-controlled infusion. Anaesthesia.

[16] Doenicke AW, Roizen MF, Rau J, Kellermann W, Babl J (1996). Reducing pain during propofol injection: the role of the solvent. Anesth Analg.

[17] Deckelbaum RJ, Hamilton JA, Moser A, Bengtsson-Olivecrona G, Butbul E, Carpentier YA (1990). Medium-chain versus long-chain triacylglycerol emulsion hydrolysis by lipoprotein lipase and hepatic lipase: implications for the mechanisms of lipase action. Biochemistry.

